# Comparison of 10-Day Course of Triple Therapy Versus 14-Day Course for Eradication of *Helicobacter pylori* Infection in an Indonesian Population: Double-Blinded Randomized Clinical Trial

**DOI:** 10.31557/APJCP.2020.21.1.19

**Published:** 2020

**Authors:** Ryan Herardi, Ari Fahrial Syam, Marcellus Simadibrata, Siti Setiati, Nikko Darnindro, Murdani Abdullah, Dadang Makmun

**Affiliations:** 1 *Department of Internal Medicine, *; 2 *Division of Gastroenterology, *; 3 *Clinical Epidemiology Unit, Department of Internal Medicine, Faculty of Medicine, Universitas Indonesia, Cipto Mangunkusumo Hospital, *; 4 *Cilincing District General Hospital, Jakarta, Indonesia. *

**Keywords:** Helicobacter pylori, duration, triple therapy, eradication, Indonesian population

## Abstract

**Objective:**

The aim of this study was to compare the effectiveness of 10-day course of triple therapy versus a 14-day course in the treatment of *H.pylori* infection in an Indonesian population.

**Methods:**

A double-blinded randomized clinical trial was included patients, Indonesian population, with *H.pylori* infection conducted in Cipto Mangunkusumo Hospital, Jakarta; Cilincing District General Hospital, Jakarta; and West Nusa Tenggara General Hospital, Mataram, during October 2016 - April 2017. Patients were randomized to be given triple therapy as Rabeprazole 20 mg, Amoxicillin 1,000 mg, and Clarithromycin 500 mg twice daily, for 14 days or 10 days plus 4 days placebo. Eradication was evaluated with UBT at least 4 weeks after completion the therapy.

**Results:**

A total of 75 patients (38 in the 14-day group and 37 in the 10-day group) were included to the study. In the intention-to-threat analysis, eradication rate was 67.6% (95% CI. 52.5%-82.6%) for the 10-day group versus 86.8% (95% CI. 76.0%-97.5%) for the 14-day group (p = 0.046), whereas per protocol analysis obtained 73.5% (95% CI. 58.6%-88.3%) for the 10-day versus 91.9% (95% CI. 84.1%-99.6%) in the 14-day group (p = 0.039). Adverse events were not significantly different between the two groups.

**Conclusion:**

A 14-day course was more effective than 10-day course of triple therapy as first-line for eradication of H.pylori infection in an Indonesian population.

## Introduction

*Helicobacter pylori* (*H.pylori*) infects a large proportion of the world’s population. Most affected individuals are asymptomatic. However, 15-20% of the case will be related peptic ulcers and 1-4% will grow as gastric malignancy. The eradication of *H.pylori* infection is one of the successful keys of peptic ulcer therapy and prevention of gastric malignancy. (Atherton and Blaser, 2013).

Nowadays, American College of Gastroenterology (ACG), Asia Pacific of Gastroenterology (APAGE), World Gastroenterology Organization (WGO), and The Indonesian Society of Gastroenterology (Perkumpulan Gastroenterologi Indonesia / PGI) recommend triple therapy, consist of proton pump inhibitor, Amoxicillin, and Clarithromycin, for the first-line therapy of H. pylori infection. Yet, questions remain regarding the optimal duration of triple therapy. For Asia, APAGE and PGI recommend triple therapy for 7-14 days. (Chey and Wong, 2007; Fock et al., 2009; Hunt et al., 2010; Syam et al., 2017). 

Successfulness of H. pylori eradication with the 7-day course triple therapy is declining. A Meta-analysis by Li et al., (2015) showed that longer than 7-day triple therapy course generates 73% eradication rate, while 10-to-14-day course generates 81%. Other meta-analysis conducted by Yuan et al, (2013) also indicated that the prolonging therapy is related with better eradications rates, whereas proportion of eradication rates of the 7-day, 10-day and 14-day course triple therapies were 76%, 80% and 86%.

Studies comparing directly between 10-day and 14-day course of triple therapy were limited and debaTable. A study by Fennerty et al., (1998), there was no difference in H. pylori eradication rates between 10-day and 14-day course triple therapy. Another study by Chen and Fallone (2015), obtained different eradication rates of *H.pylori* infection between two groups, 82.7% for 14-day course and 45.2% for 10-day course. Thus, this study compares the effectiveness between 10-day course and 14-day course triple therapy in eradication of *H.pylori* infection in an Indonesian population.

## Materials and Methods

*Study subjects and Materials*

A prospective double-blinded randomized clinical trial was conducted from October 2016 to June 2017 in Cipto Mangunkusumo Hospital, Jakarta; Cilincing District General Hospital, Jakarta; and West Nusa Tenggara General Hospital, Mataram, Indonesia. The following criteria applied to the patients to be admitted to the study : Indonesian people, aged over 18 years, signed informed consent, and positive for *Helicobacter pylori* infection from UBT or histopathology biopsy from esophagoduodenocopy. We excluded patients obtained previous *H.pylori* therapy, abnormal ALT, arrhythmia, prolonged QT, and pregnant or breastfeeding woman.

*According to the two independent sample size formula as follows*

with the proportion 63.6% and expected proportion 91.4% (from previous studies), we obtained that minimum sample is 34 patients for each group.11,12

Patients did not use proton pump inhibitor or antibiotics for 2 weeks before UBT or esophagoduodenoscopy. Patients with positive results were randomized using Microsoft Excell 2015 ®, into either group A or group B. Patients and physicians did not know where the patients were given triple therapy as Rabeprazole 20 mg, Amoxicillin 1,000 mg, and Clarithromycin 500 mg twice daily for 14 days or Rabeprazole 20 mg, Amoxicillin 1,000 mg, and Clarithromycin 500 mg twice daily 10 days plus 4 days placebo. Only the pharmacist knew the allocation of drugs given to the patients group A and group B (blinding process). We also documented patients’ demographics (sex, age, ethnic, level of education, and salary), smoking habits, nutritional status, and comorbid disease. Patients’ sex and age were based on their ID Card at the time the study was done. Patients’ ethnic was asked to them (Javanese, Sundanese, Buginese, Batak, Balinese, Betawi, Malay, and others). Level of educational was the patient’s last education (Low for not graduated or Elementary School; Medium for Junior High School or Senior High School; High : Diploma, Undergraduate, Graduate, or Post-graduate). Salary was the patient’s monthly income (Below or Above the 2016 Regional Minimum Income. The 2016 Regional Minimum Income for Jakarta was IDR. 3,100,000. (about USD 235.00) per month and Mataram was IDR 1,482,950 (about USD 115.00) per month. Smoking habits was asked to the patient at the time the study was done. Nutritional status was determined by patient’s Body Mass Index (BMI) (underweight : BMI less than 18.5 kg/m^2^; normal : BMI 18.5-22.9 kg/m^2^; Overweight : BMI 23.0-25.0 kg/m^2^; and Obesity : BMI above 25.0 kg/m^2^). Comorbid disease was based on medical record (Type 2 Diabetes Mellitus (DM); Hypertension; Dyslipidemia; Coronary Artery Disease; Ashtma; Osteoarthritis; and other diseases). Through the research, patients obtained appropriate medical therapy for their comorbid disease. All patients were carefully monitored regarding their compliance and adverse effects (allergic, taste perversion, epigastric pain, headache, nausea, vomiting, and others). Patients were underwent UBT to evaluate the successfulness of eradication, at least 4 weeks after finishing the treatment. In the end of the study, we opened the label and got that group A was 14-day course triple therapy and group B was 10-day course triple therapy.

This study protocol has approved by The Ethics Committee of the Faculty of Medicine, Universitas Indonesia, Jakarta with protocol number : 16-10-311 and registered at www.clinicaltrials.gov with ID number: NCT03134378.

*Statistical analysis*

Continous variables were stated as mean, while 95% Confidental Intervals were calculated for categorical variables using the standard normal approximation of the binomial distribution. Chi-square test (*X*^2^) or Fisher’s exact test was performed to prove the significant differences of proportion of eradication between the 14-day course triple therapy and 10-day course triple therapy. Statistical analysis was performed using both an intention-to-treat (ITT) analysis, in which a patients with missing eradication data was considered to be a treatment failure, and a per-protocol (PP) analysis, in which a patients with missing eradication data was excluded from calculation. Statistical analysis was performed using the SPSS^® ^software package version 22.0. 

## Results

**Figure 1:**
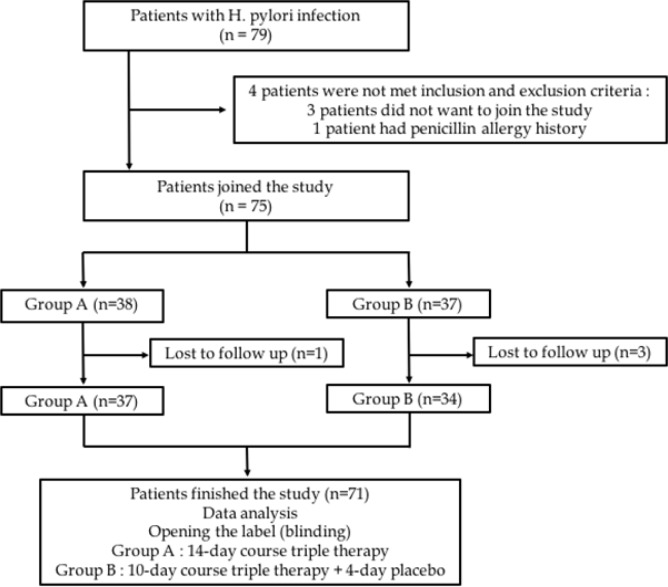
Research Flow Diagram

A total of 79 patients acquired positive *H.pylori* infection. Three patients did not sign the informed consent and 1 patient had history of penicillin allergic, thus 75 patients met the criteria for study. The randomized process created 38 patients in group A and 37 patients in group B. At the end of, 4 patients (1 in the group A and 3 in the group B) were lost to follow up. The physicians interpreting the results were blinded to the treatment allocation. Figure 1. depictes the research flow. All baseline characteristics were similar for the two treatment groups with respect to demographics (sex, age, ethnic, level of educational, and salary), smoking habits, nutritional status, and comorbid disease. Table 1 describes baseline characteristics between those two groups.

Using ITT analysis, we obtained that the group A eradication rate was 86.8% (95% CI. 76.0%-97.5%) while the group B eradication rate was 67.6% (95% CI. 52.5%-82.6%), and statistically significant (p = 0.046; Chi-Square test). The ITT analysis is described in the Table 2. Expending PP analysis, we obtained that the group A eradication rate was 91.9% (95% CI. 84.1%-99.6%), whereas the group B eradication rate was 73.5% (95% CI. 58.6%-88.3%), and statistically significant (p = 0.039; Chi-square test). The PP analysis is depicted in the Table 3. Adverse events (taste pervesion, nausea, vomiting, headache, fatigue, and diarrhea) were not significantly different between the two groups. Monitoring the adverse effects, we concluded that the most frequent of adverse effects is perversion taste. Patients felt flavorless taste when drinking mineral water. We find that the adverse effects between two groups tend to be the same. Table 4 reports the adverse effects.

**Table 1:** Baseline Characteristics between Two Groups

Variable	14-day *Triple Therapy *(n=38)	10-day *Triple Therapy *+ 4-day Placebo (n=37)
Sex; n (%)		
Male	17 (44.7)	12 (32.4)
Female	21 (55.3)	25 (67.6)
Age; years		
Mean	44.05	45.08
	(95% CI : 39.26-48.84)	(95% CI : 39.94-50.23)
Classification of age; years (%)		
18-30	9 (23.7)	7 (18.9)
30-40	6 (15.8)	8 (21.6)
40-50	7 (18.4)	9 (24.3)
50-60	11 (28.9)	5 (13.5)
> 60	5 (13.2)	8 (21.6)
Ethnic; n (%)		
Bugisnese	11 (28.9)	11 (29.7)
Batak	8 (21.1)	7 (18.9)
Javanese	4 (10.5)	5 (13.5)
Betawi	4 (10.5)	3 (8.1)
Sundanese	3 (7.9)	3 (8.1)
Sasak	3 (7.9)	3 (8.1)
Others	5 (13.1)	5 (13.5)
Level of Education; n (%)		
Low	9 (23.7)	10 (27.0)
Medium	19 (50.0)	18 (48.6)
High	10 (26.3)	9 (24.3)
Salary / Monthly income; n (%)		
Below Regional Min Income	24 (63.2)	25 (67.6)
Above Regional Min Income	14 (36.8)	12 (32.4)
Smoking habits; n (%)		
Yes	8 (21.1)	8 (21.6)
No	30 (78.9)	29 (78.4)
Nutritional Status; n (%)		
Underweight	5 (13.2)	4 (10.8)
Normal	14 (36.8)	17 (45.9)
Overweight	7 (18.4)	7 (18.9)
Obesity	12 (31.6)	9 (24.3)
Comorbid Disease; n (%)		
Type 2 DM	4 (10.5)	1 (2.7)
Hypertension	6 (15.8)	2 (5.4)
Dyslipidemia	1 (2.6)	3 (8.1)
Coronary Artery Disease	2 (5.3)	1 (2.7)
Asthma	1 (2.6)	1 (2.7)
Osteoarthritis	2 (5.3)	1 (2.7)

**Table 2:** Intention-to-Treat Analysis

	Proportion of eradication				
Research Group	Successful		Failed/ Lost-to-follow up		p *
	n	%	n	%	
14-day *Triple Therapy *(n=38)	33	86.8	5	13.2	0.046
10-day* Triple Therapy * + 4-day Placebo (n=37)	25	67.6	12	32.4	
Total	58	77.3	17	22.7	

* p, Chi-square test

**Table 3:** Per-Protocol Analysis

Research Group	Proportion of Eradication				
	Successful		Failed		p *
	n	%	n	%	
14-day *Triple Therapy* (n=37)	34	91.9	3	8.1	0.039
10-day *Triple Therapy* + 4-day Placebo (n=34)	25	73.5	9	26.5	
Total	59	83.1	12	16.9	

* p, Chi-square test

**Table 4:** Adverse Effects During Triple Therapy Given to Patients

Adverse Effect; n (%)	14-day Triple Therapy (n=38)	10-day Triple Therapy + 4-day placebo (n=37)
taste pervesion	9 (23.7)	7 (18.9)
nausea	7 (18.9)	7 (19.4)
vomiting	1 (2.6)	1 (2.7)
headache	0 (0)	1 (2.7)
fatigue	2 (5.3)	3 (8.1)
diarrhea	0 (0)	2 (5.4)

## Discussion

The proportion of female patients were higher than other study. Kawakubo et al., (2016), showed that complaints in the upper gastrointestinal symptoms were more frequent in female than male. In both groups, the number of female patients were similar. *H.pylori* infected all ages, related with previous studies conducted by Goto et al., (2016) and Darnindro et al., (2015). Many studies also showed that *H.pylori* infection spread across all ages. Furthermore, it was uncommon to find the infection in the children who inherited the infection from their parents. We obtain comparable age between both groups.

Bugisnese and Batak were the most ethnics infected by *H.pylori* in this research. This finding also confirmed the findings from Goto et al., (2016) and Darnindro et al., (2015). in the previous study in Indonesia. Goto et al., (2016), noticed that eating using hand was more frequent in Buginese and Batak than other ethnics, thus they proned to the *H.pylori* infection. We found that *H.pylori* infection accross all of the education levels. This was related with the study from Darnindro et al., (2015), showing that *H.pylori* infection spreads across all education levels. 

We expected salary was a risk factor of the *H.pylori* infection. Most of our patients had monthly income below 2016 Regional Minimum Income. According to Bruce (2008), education level and monthly income were risk factors causing the *H.pylori* infection. Indeed, this hypothesis was debaTable because some studies explained that low education level and low income were associated with the high proprotion of *H.pylori* infection, while other studies proved no association between low education and income levels with *H.pylori* infection. Nutritional level was also considered as a success factor of *H.pylori* infection. Abdullahi et al., (2008), showed that overweight and obesity patients had lower successful for eradication of *H.pylori* infection. Our study has similar proportion of nutritional status in the both group.16,17

Smoking was an important variable in *H.pylori* infection. The study conducted by Suzuki et al., (2016), exhibited that smoking could decrease the successful for eradication of *H.pylori* infection. Our study has similar proportion of smoking in the both group. We also suggested that the patients reduced their smoking frequency during the research period (Syam et al., 2017).

With ITT analysis, we found that the eradication rate of the *H.pylori* infection in the 14-days course triple therapy was 86.6%, higher than 10-days course 67.6% (p=0.046; Chi-square test). Our finding was consistent with Chen and Fallone (2015), whereas the eradication rate of the *H.pylori* infection using 14-days course triple therapy was 82.7%, higher than 10-days course which was only 45.2%. Using per-protocol analysis, we found that the eradication rate of the *H.pylori* infection in the 14-days course triple therapy is 91.9%, higher than 10-days course triple therapy which is only 73.% (p = 0.039). Our finding in the difference is consistent with Chen and Fallone (2015). whereas the eradication rate of the *H.pylori* infection using 14-days course triple therapy is 91.5%, and 10-day course was only 63.6%. Our study is different from Chen and Fallone (2015), because our design study was double-blinded clinical trials and we have only 5.3 percent lost-fo-follow up respondents, and we used Rabeprazole as proton pump inhibitor.

Fennerty et al., (1998), has different conclusion from our study. They concluded that there is no significant different between 10-day course and 14-day course triple therapy. We should note that their study was conducted 19 years ago, when the research on *H.pylori* infection had just been started and antibiotical resistance were lower than nowadays. Furthermore, they used Lansoprazole as the proton pump inhibitor.

Comparing with the study from Li et al., (2015), it was clearly shown that our research was in line. We could see in their analysis demonstrating the increment of the eradication rate of the *H.pylori* infection in the prolonging triple therapy group. However, Li et al., (2015) demonstrate only 6% difference in the proportion between 14-day course and 10-day course triple therapy. This findings were thought because of very diverse ethnics, geographical locations, periods and proton pump inhibitor used in their research. Furthermore, their results were obtained from additional analysis, instead of direct comparisons between 14-day course and 10-day course triple therapy.

Our study justified recent recommendation from American College of Gastroenterology (ACG) on February 2017. ACG showed that the first line therapy that has been used and approved by United States Food and Drug Administration (FDA) was triple therapy using proton pump inhibitor, Clarithromycin 500 mg, and Amoxicillin 1,000 mg twice daily for 14 days. However, we should note that the patients had to be asked regarding their previous macrolide history and the Clarithromycin resistance was less than 15%. Therefore, APAGE and PGI need to issue a new recommendation on the duration of triple therapy for *H.pylori* infection eradication in Asia, especially in Indonesia (Chey et al., 2017).

According to the theory, the duration of triple therapy was related to the eradication rate. This was in line with previous research documenting that 3-day course and 5-day course triple therapies have been abandoned. Furthermore, 7-day course triple therapy was no longer recommended due to low eradication rate. However, we might ignore the theory because some of the determinants causing the failure for eradication of the *H.pylori* infection are the antibiotics resistance and low compliance of the patient. (Chey and Wong, 2007; Fock et al., 2009; Hunt et al., 2010; Syam et al., 2017).

The bacteria could still live in the gaster that was acid by producing urease enzymes that dissolve urea becoming ammonia. The ammonia would be used to protect the bacteria from the acid. *H.pylori* can be bound in all parts of gaster (fundus, antrum or corpus). If the lumen of the gaster was acid, the bacteria tent to be found in antrum area (far from antibiotics’ reach). If the gastric acid was minimized, the bacteria tent to be found in the corpus. Thus, the bacteria could be easily reached by the antibiotics (Kusters et al., 2006).

The bacteria could naturally defend themselves by transforming them from spiral to small coccoid. The small coccoid did not degenerate. Thus, this causes difficulties in the eradication. The small coccoid would be back to spiral form in the next 1-2 weeks. Therefore, the 1-2 weeks period became a good reference for the duration of triple therapy for the eradication of *Helicobacter pylori* infection. If the therapy was conducted for the period equals or less than a week, the bacteria might defend themselvers by the trasformation process. If the therapy was conducted for the 10-day period, the drugs would attack the bacteria only in few days (and then the bacteria would defend in the remaining days). If the therapy was conducted for the 14-day period, the drugs would “fight” the bacteria in the longer period. Thus, the drugs could still attack the bacteria when the they have transformed back into original form. The clarithromycin resistance was 9.1% in Indonesia, but the prevalence of clarithromycin resistance in Java island (Jakarta is a part of Java island) was 21.4%. Thus, it was thougt that triple therapy should be given with a longer duration in this population (Kusters et al., 2006; Miftahussurur et al., 2016).

In conclusion, the 14-day course was better than 10-day course triple therapy for eradication of the *H.pylori* infection in an Indonesian population. There were no differences in adverse effects that occurred in both groups.
